# Caecal infusion of the short‐chain fatty acid propionate affects the microbiota and expression of inflammatory cytokines in the colon in a fistula pig model

**DOI:** 10.1111/1751-7915.13282

**Published:** 2018-06-01

**Authors:** Yanan Zhang, Kaifan Yu, Huizi Chen, Yong Su, Weiyun Zhu

**Affiliations:** ^1^ Laboratory of Gastrointestinal Microbiology Jiangsu Key Laboratory of Gastrointestinal Nutrition and Animal Health College of Animal Science and Technology Nanjing Agricultural University Nanjing 210095 China; ^2^ National Center for International Research on Animal Gut Nutrition Nanjing Agricultural University Nanjing 210095 China

## Abstract

Short‐chain fatty acids (SCFAs), particularly butyrate, are known to suppress inflammation, and regulate the gut bacterial ecology. However, little is known about propionate. We report here that propionate infusion in the caecum dramatically affected the structure of colonic microbiota of pigs based on 16s rRNA high‐throughput sequencing. Sixteen pig models were perfused with saline or sodium propionate by a fistula in the caecum. At d 28, all pigs were slaughtered for analysing bacterial metabolites, colonic microbiota and the expression of genes related to inflammation. The results showed that caecal infusion of sodium propionate increased the concentration of propionate and decreased the butyrate concentration in colonic content. For biogenic amines, the tyramine concentration was increased, while the concentration of cadaverine was decreased by infusion of sodium propionate. Furthermore, at the level of phylum, propionate increased the abundance of *Bacteroidetes* and reduced the abundance of *Firmicutes*. *Prevotella* and *Bacteroides* counts were increased, while *Turicibacter* abundance was decreased at the level of genus. Real‐time qPCR showed that the expression of NF‐κB and IL‐18 was upregulated by propionate infusion, whereas no significant differences were observed for the expression of other genes related to inflammatory processes. Taken together, these results provide a new evidence for the role of short‐chain fatty acid propionate on the composition of microbial community and inflammatory cytokines.

## Introduction

In recent years, there has been a widespread concern about protecting against the intestinal diseases and improving intestinal health. The intestines not only play a part in the digestion and absorption, but also play a part in the immune system function. The development of the intestinal mucosa immune system is promoted by the early gut microbial colonization (Maslowski and Mackay, 2011; Hansen *et al*., 2012). Previous studies have reported that intestinal microbes and microbial metabolites, in particularly short‐chain fatty acids (SCFAs), were associated with genes related to inflammation expression of intestinal mucosa (Mu *et al*., 2016; Zhang *et al*., 2017).

Short‐chain fatty acids, predominantly including acetate, propionate and butyrate, which are the major products of bacterial fermentation of indigestible carbohydrates, exert a variety of physiologic effects. SCFAs are the main source of energy for the ruminant, as well as provide energy for the intestinal epithelial tissue of mono‐gastric animals to facilitate the proliferation and differentiation of epithelial cells (Scheppach, 1994; Hamer *et al*., 2008; Furusawa *et al*., 2013). In addition, SCFAs serve as molecular signals and play a positive role in anti‐inflammation, inflammation bowel disease (IBD) and anticarcinogenesis (Singh *et al*., 2014). Anti‐inflammatory functions of SCFAs have been extensively investigated, particularly butyrate, which has been shown to have an important role in inhibiting inflammatory process. Maslowski and colleagues (2009) and Xu and colleagues (2016a) have shown that butyrate upregulated the expression of anti‐inflammatory cytokines and downregulated proinflammatory agents in the intestinal epithelium. In addition to acting as signalling molecules, SCFAs also maintain the balance of intestine microbial ecosystem, which contributes to gut immune development (Lee and Mazmanian, 2010; Chung *et al*., 2012; Kamada *et al*., 2013). Several studies have demonstrated that butyrate regulates the microbial composition (Xu *et al*., 2016a), and affects the ecological structure and metabolic activity of the gut microbiota (Castillo *et al*., 2006; Biagi *et al*., 2007). Collectively, this evidence suggests that SCFAs indirectly affect the development of intestinal immune system by modulating the homeostasis of microbiota. However, with regard to the anti‐inflammatory effects of SCFAs, most studies have focused more attention on butyrate, and the impact of propionate on intestinal inflammation has not been given much attention.

Nuclear factor‐κB (NF‐κB) is a nuclear transcription factor that regulates gene expression of apoptosis, inflammation and autoimmune disease. Histone deacetylase (HDAC), a class of protease, modifies chromosome structure to regulate the expression of cytokines, including IL‐6, IL‐8 and IL‐18 (Xu *et al*., 2016a). The activation of NF‐κB or HDAC is thought to promote proinflammatory cytokine transcription and induce the inflammatory responses (Saleh and Trinchieri, 2011; Guo *et al*., 2013). Butyrate has been reported to inhibit activation of HDAC and NF‐κB; Butyrate also reduces the production of inflammatory cytokines (Vinolo *et al*., 2011; Xu *et al*., 2016a). However, it is not clear whether propionate also affects the NF‐κB and HDAC pathways that regulate the transcription of cytokines.

Thus, we hypothesize that propionate will influence the composition of the microbial community and the expression of inflammatory‐related genes in the colonic epithelial mucosa. We utilized a pig model to explore the effects of infusion with propionate on the composition of the colonic microbiota and the gene expression of inflammatory cytokines of the colonic epithelium.

## Results

### Microbial metabolites of colonic content

**Figure 1:**
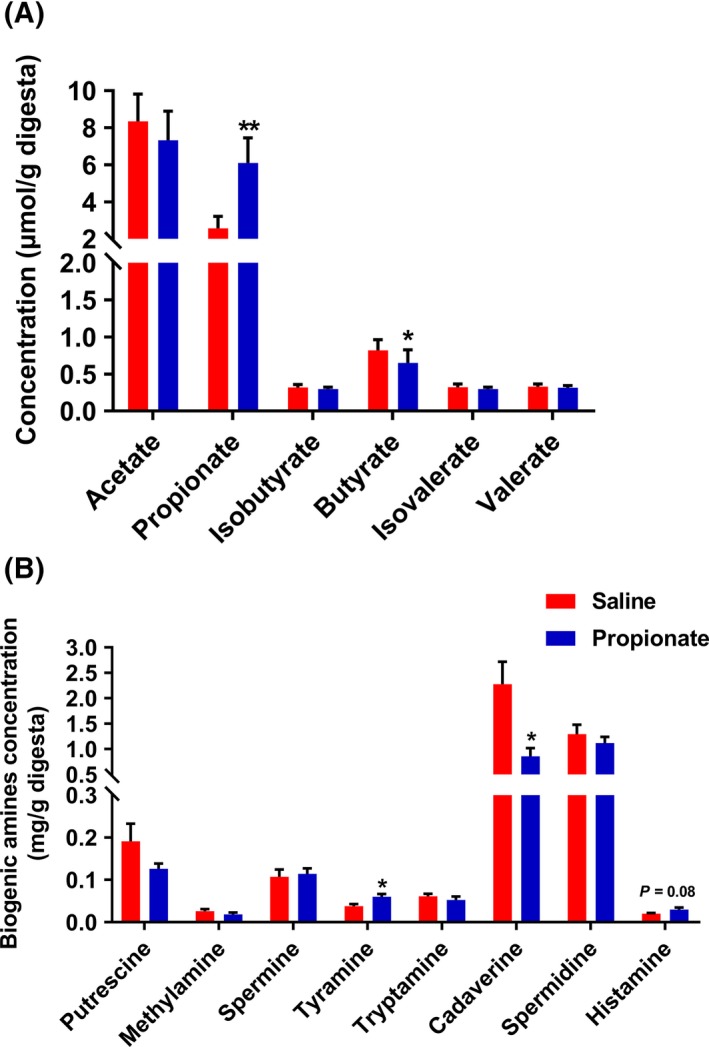
The effects of infusion propionate on SCFA (A) and biogenic amines (B) in colon content.

We evaluated microbial metabolites including SCFAs and biogenic amines. As shown in Fig. 1A, caecal infusion of sodium propionate significantly increased the concentration of propionate (*P *<* *0.05). Interestingly, the concentration of butyrate was significantly decreased in the treatment group (*P *<* *0.05) compared with control. There were no significant differences in the concentrations of acetate, isobutyrate, isovalerate and valerate between the treatment and control groups (*P *>* *0.05). Furthermore, caecal infusion of sodium propionate significantly increased the concentration of tyramine and reduced the concentration of cadaverine in the colon (*P *<* *0.05). The histamine concentration has a tendency to increase (*P *=* *0.08). Compared with the control group, caecal infusion of sodium propionate had no significant effects on the concentrations of methylamine, putrescine, spermine, tryptamine and spermidine (*P *>* *0.05) (Fig. 1B). These results suggest that caecal infusion of sodium propionate affects microbial fermentation of carbohydrates and protein in the colon.

### Bacterial community of colonic content

**Figure 2:**
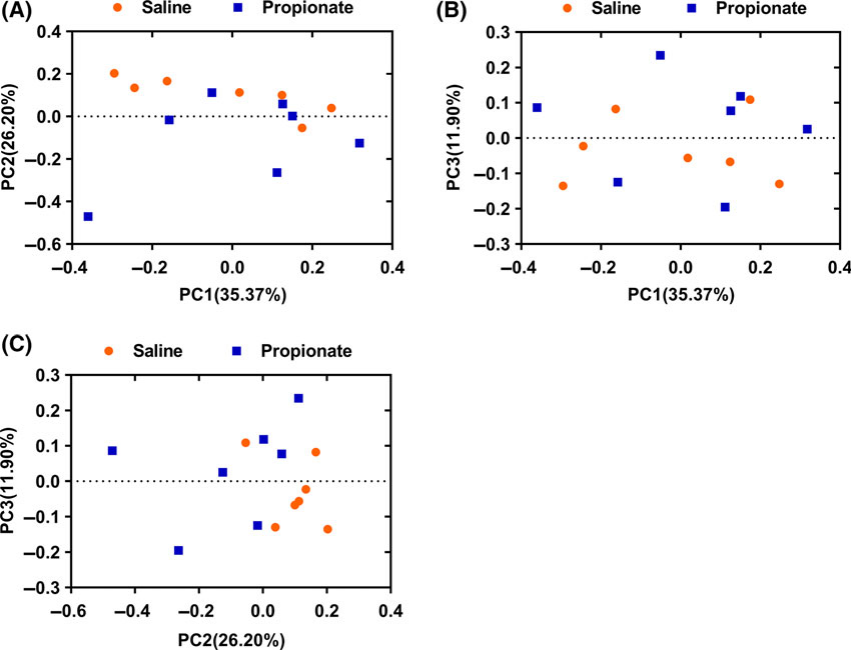
Colonic digesta bacterial community analysis of pigs by pyrosequencing analysis. Principal coordinates analysis plot based on the relative abundance of OTUs using MOTHUR software. The graphs on a two‐dimensional array of (A) PC1 and PC2, (B) PC1 and PC3 and (C) PC2 and PC3 were shown respectively. PC1, principal coordinate 1; PC2, principal coordinate 2; PC3, principal coordinate 3.

Next, we determined the composition of the colonic microbiota using 16S rRNA high‐throughput sequencing. We obtained 337 263 sequence reads meeting the quality criteria from all the samples with an average of 24 090 reads per pig. The average length of the quality sequence was 456 bp. The rarefaction curves tended to approach a saturation plateau (Fig. S1), indicating that there were sufficient sequences to reflect diversity and abundance of bacterial community. At the taxonomic level, the Simpson and Shannon diversity index suggested no significant difference between the two groups, while Chao index of species richness was increased (*P *<* *0.05) and the abundance‐based coverage estimator (ACE) increased (*P *=* *0.07) in the SP group (Table 1). Moreover, PCA showed that the microbial communities of pigs in the two groups were not significantly different (Fig. 2A–C).

**Table 1:** Effects of infusion sodium propionate on the diversity of colonic microbial community at the 3% dissimilarity level

Item	Saline	Propionate	*P*‐value
OTUs	608 ± 42	692 ± 23	0.104
Coverage (%)	99.03 ± 0.06	98.92 ± 0.04	0.123
Diversity indices			
Simpson	0.07 ± 0.01	0.11 ± 0.02	0.204
Shannon	3.77 ± 0.08	3.69 ± 0.14	0.624
Richness			
Chao	907.35 ± 56.28	1054.75 ± 33.23	0.044
Ace	1089.48 ± 71.23	1251.44 ± 31.81	0.070

Ace, abundance‐based coverage estimator; OTUs, Operational taxonomic units.

**Figure 3:**
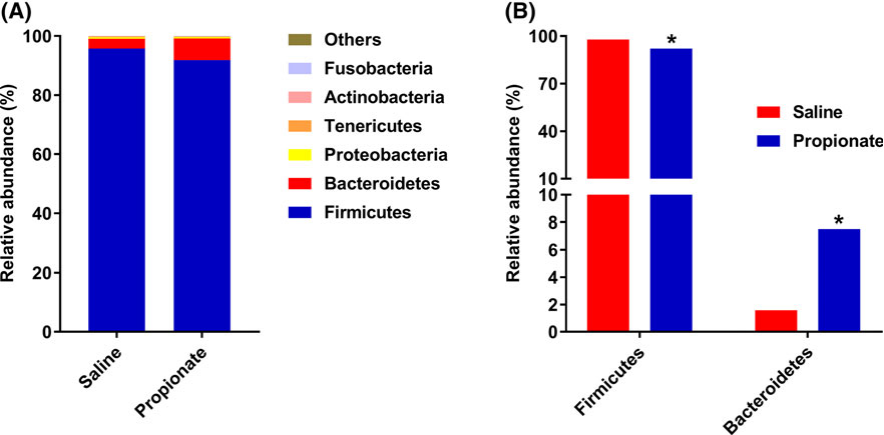
Relative abundance of reads at phylum‐level (A) and significantly different phyla (B) (Mann‐Whitney U‐*test*, Values are medians). * *P *<* *0.05, ** *P *<* *0.01.

**Figure 4:**
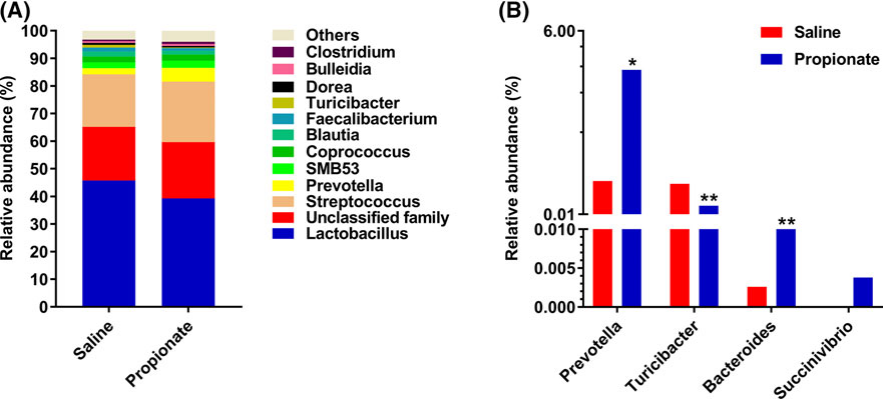
Relative abundance of reads at genus‐level (A) and significantly different genera (B) (Mann–Whitney *U*‐*test*, Values are medians). * *P *<* *0.05, ** *P *<* *0.01.

**Figure 5:**
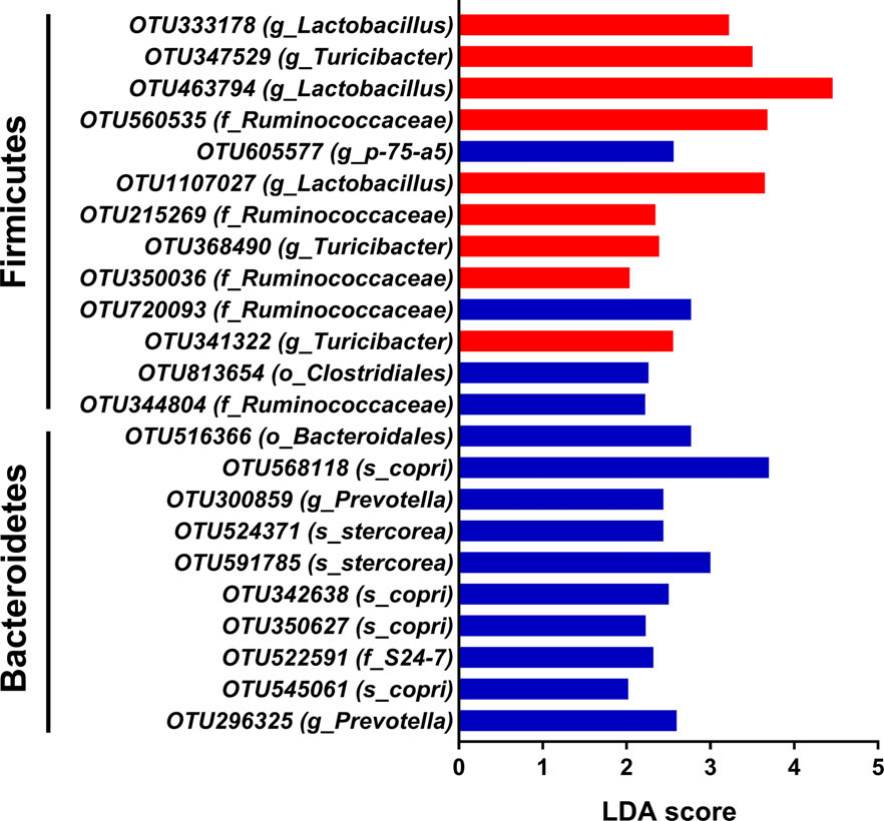
The significantly different OTUs of bacterial community of the control group were presented by LDA. LDA score of the significantly different OTUs was set as 2.

At the phylum level, we identified six phyla: *Firmicutes, Bacteroidetes, Proteobacteria, Tenericutes, Actinobacteria,* and *Fusobacteria*. Among these phyla, *Firmicutes* was the most predominant phyla in bacterial community of colonic content, and accounted for 95.8% and 91.9% in the control group and sodium propionate (SP) group respectively. *Bacteroidetes* accounted for 3.3% in the control group and 7.3% in the SP group, which was the second most dominant phylum (Fig. 3A). Furthermore, we found that infusion of propionate significantly increased the abundance of *Bacteroidetes* (*P *<* *0.05), and decreased the abundance of *Firmicutes* (*P *<* *0.05) (Fig. 3B). Genus‐level analysis indicated that there were eleven primary genera. *Lactobacillus, Streptococcus* and *Prevotella* were found to be the dominant genera in the colonic digesta (Fig. 4A). Compared with the control group, the SP group had higher abundance of *Prevotella* and *Bacteroides* (*P *<* *0.05), and had a tendency for higher abundance of *Succinivibrio* (*P *=* *0.073). The relative abundance of *Turicibacter* was significantly decreased in the SP group (*P *<* *0.05) (Fig. 4B). Moreover, LDA analyses also confirmed that the phylum *Firmicutes* and *Bacteroidetes* counts were significantly altered by infusion of propionate (Fig. 5). At the OTUs level, infusion of propionate significantly increased the abundance of *Prevotella‐*related OTU, but decreased the abundance of *Lactobacillus‐*, and *Turicibacter‐*related OTU in the colonic microbial community (Table S1).

### Gene expression in colonic mucosa

**Figure 6:**
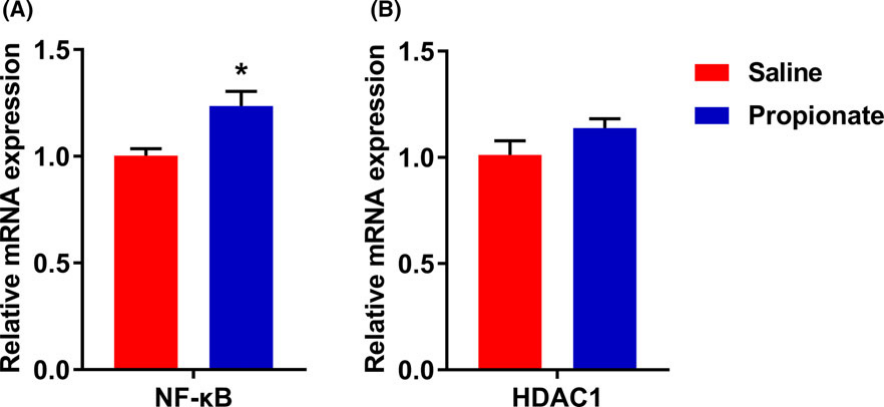
The effects of infusion propionate on gene expression of NF‐κB (A) and HDAC1 (B). Values are mean ± SEM. * *P *<* *0.05.

**Figure 7:**
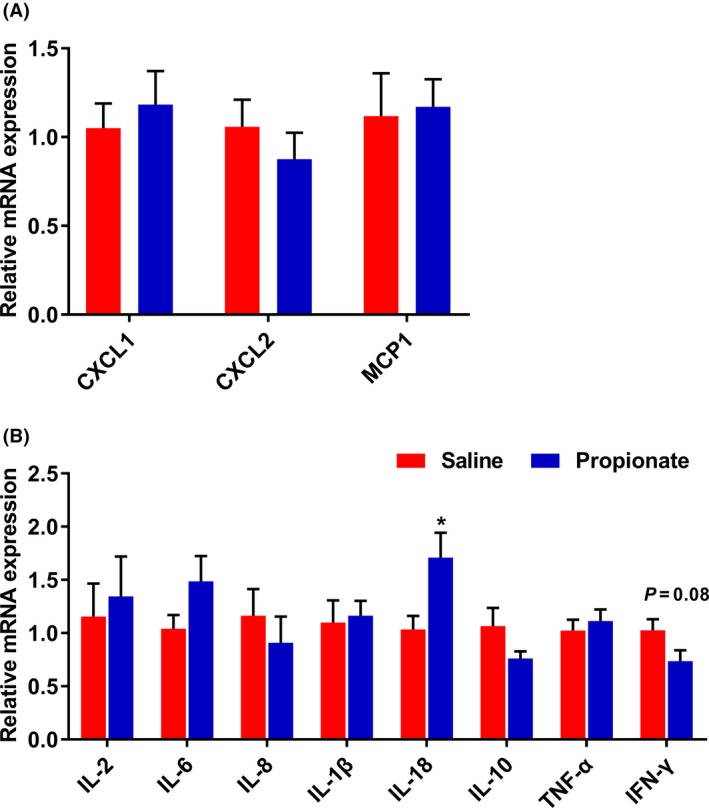
The effects of infusion propionate on gene expression of (A) chemotactic factor and (B) inflammation. Values are mean ± SEM. * *P *<* *0.05.

To assess the effects of propionate infusion on immune response, we measured the expression of inflammation‐related genes. As shown in Fig. 6, the mRNA expression of NF‐κB was significantly upregulated (*P *<* *0.05) in the SP group, while there was no significant influence on the expression of HDAC1 between two groups. Furthermore, infusion of propionate did not affect the expression of chemokines (Fig. 7A). We also found no significant differences in the expression of inflammatory cytokines, except for IL‐18. Compared with the control group, the mRNA level of IL‐18 was higher in the SP group (*P *<* *0.05). Infusion of propionate also had a tendency to decrease the mRNA expression of IFN‐γ (*P *=* *0.08) (Fig. 7B).

## Discussion

In this study, we used a fistula pig model to evaluate the effects of propionate on the colonic microbial composition and the expression of inflammatory cytokines in the colonic mucosa. The results revealed that propionate altered the concentration of bacterial metabolites, the composition of the colonic microbiota, and upregulated NF‐κB expression in the colonic epithelium, but had no influence on the expression of inflammatory cytokines, except for IL‐18. These findings provide a new understanding that propionate plays a role in modulating colonic microbiota and the expression of genes related to inflammation in the colonic epithelium.

Alterations of microbial metabolites affect the gut microbiota. In this study, we showed that infusion of propionate increased the richness estimator (Chao), and altered the relative abundance of several bacteria. At the phylum level, the abundance of *Firmicutes* and *Bacteroidetes* were markedly altered by infusion of propionate. The increased *Bacteroidetes* and decreased *Firmicutes* may contribute to the modified concentration of SCFAs. Previous studies indicated that the bacteria in *Bacteroidetes* phylum are the major acetate and propionate producers, and the bacteria in *Firmicutes* phylum are the major butyrate producers (Louis *et al*., 2007; Flint *et al*., 2012). SCFAs are major products of non‐digestible carbohydrate fermentation by the microbiota in the hindgut, which are made up 95% by acetate, propionate and butyrate (Macia *et al*., 2015). In the current study, following infusion of propionate, the concentration of propionate was increased, interestingly, whereas the colonic butyrate concentration decreased. It is well‐known that SCFAs provide energy for the intestinal epithelium, therefore, these findings suggest that infused propionate may replace a part of butyrate to serve as an energy source for intestinal epithelium (Hague *et al*., 1996; Singh *et al*., 1997). The infusion of propionate also significantly increased the length of colon (data not shown). Furthermore, the colonic bacterial fermentation of protein was affected by perfusing propionate in which propionate treatment group had increased tyramine concentration and decreased cadaverine concentration. Taken together, these findings indicate that the pattern of microbial fermentation in the colon is altered by propionate.

Alterations in the ratio of *Firmicutes* and *Bacteroidetes* are associated with intestinal health. In recent years, evidence suggests that the mammalian gastrointestinal (GI) tract is home to a complex and immense community of microorganisms that are engaged in a dynamic interaction with host intestinal health (Peterson *et al*., 2008; Cerf‐Bensussan and Gaboriau‐Routhiau, 2010; Kamada *et al*., 2013). Therefore, breakdown of the gut microbial homeostasis and loss of diversity in early life (i.e., childhood) can dramatically increase the risk of GI diseases (Tao *et al*., 2015; Dou *et al*., 2017). The microbiota composition, which is beneficial to the gut health, has been extensively investigated. A previous report pointed out that, in a health state, the dominant bacterial phyla are the *Firmicutes*,* Bacteroidetes* and *Actinobacteria* (Tremaroli and Backhed, 2012). Compared with children living in urban areas in Europe, the composition of the foecal microbiota, which had higher levels of *Bacteroidetes* and lower of *Firmicutes*, was beneficial to the intestinal health of African children living in rural areas (De Filippo *et al*., 2010). In addition, Frank and colleagues (2007) identified that the microbial diversity was significantly perturbed with a distinct depletion of *Bacteroidetes* in patients with IBD as compared to healthy people. Collectively, these findings suggest that the changes in the abundance of *Firmicutes* and *Bacteroidetes* bacterium are associated with intestinal health. In the present study, the data presented also suggest that the abundance of *Firmicutes* and *Bacteroidetes* altered by propionate favours protection of the colonic epithelium to promote gut health.

At the genus level, our study revealed *Lactobacillus* and *Streptococcus* are dominant genera. *Lactobacillus* produces hydrogen peroxide and lactic acid to inhibit the growth of pathogenic bacteria (Mikkelsen *et al*., 2004); *Streptococcus* creates an anaerobic environment favoring the establishment of other colonizers such as *Bacteroides*,* Lactobacillus* and *Bifidobacterium* (Petri *et al*., 2010). A previous research provides some evidence that the habitation of *Lactobacillus* and *Streptococcus* is prerequisite condition in maintaining the homeostasis of gut microbiota. Additionally, infusion of propionate significantly increased the abundance of *Bacteroides* and *Prevotella*, and decreased *Turicibacter* counts. A previous study indicated that the bacterial *Bacteroides* species produce a particular glycan, polysaccharides A, and colonization of germ‐free mice by the *Bacteroides* strain or administration with polysaccharides A protected against experimental IBD in mice (Maslowski and Mackay, 2011). *Turicibacter* genus, Gram‐positive bacteria in the *Firmicutes* phylum, is known to have deleterious effects in inducing mucosal injury (Lupp *et al*., 2007; Kellermayer *et al*., 2011). Thus, the increased *Bacteroides* and decreased *Turicibacter*, which were also confirmed by the results of LDA score and OTU level, are associated with gut health.

Butyrate, modulates inflammatory processes by inhibiting the activity of NF‐κB and HDAC1, and affects gene expression in colonic epithelial cells (Xu *et al*., 2016b). We also previously showed that butyrate downregulates HDAC1 and proinflammatory cytokines, and upregulates the expression of anti‐inflammatory cytokines in the gut (Xu *et al*., 2016a). Compared to butyrate, propionate exerts an equipotent effect, at suppressing the NF‐κB reporter activity, immune‐related gene expression and cytokine release *in vitro* (Tedelind *et al*., 2007). However, in the present study, we did not find an effect of propionate on genes expression related to inflammation, except for upregulation of IL‐18 and NF‐κB expression *in vivo*. IL‐18 is increased in specific pathogen free (SPF) mice treated with 200 and 300 mM acetate, and act in maintenance of epithelial integrity, repair and intestinal homeostasis (Singh *et al*., 2014; Macia *et al*., 2015), whereas it is unclear and will require further investigation to understand how propionate affects the expression of IL‐18. Moreover, using Pearson's correlation, we identified that *Streptococcus* strain was positively correlated with NF‐κB activation (data not shown). Several species of *Streptococcus* bacteria, such as *Streptococcus pyogenes* (Tsai *et al*., 2006) and group B *Streptococcus* (Vallejo *et al*., 2000), have been shown to induce the activation of NF‐κB. Based on our findings, infusion of propionate has small effect on the expression of cytokines.

In summary, the present study demonstrates that propionate alters the composition of the microbial community, which increases the benefit bacteria and decreases harmful bacteria. Infusion of propionate increases the expression of NF‐κB and IL‐18, while the expression of other genes related to inflammatory processes fail to show differences in the colonic mucosa. These findings provide a new evidence for the role of short‐chain fatty acid propionate on the regulation of the colonic microbiota and inflammatory cytokines.

## Materials and methods

The experimental design and procedures for the care and treatment of pigs were approved by the Animal Care and Use Committee of Nanjing Agricultural University (Nanjing, Jiangsu province, China), and followed the Chinese guidelines for animal welfare.

### Experimental design

Sixteen Duroc × Landrace × Large White growing barrows from a commercial farm were used. Each barrow was placed in an individual pen with *ad libitum* access to feed (a commercial diet) and water. The experimental diet consisted of 35% commercial concentrate feed and 65% corn, of which the composition and nutrient contents are shown in Table 2. After a 3 days pre‐feeding period, we surgically installed a fistula in the caecum of pigs. After 14 days, a postoperative recovery period, the growing barrows were randomly assigned to two group (about 20 kg body weight, *n* = 8 per group) and perfused with a saline (the control group) or with a sodium propionate (the SP group) solution (25 ml per time, 2 M, pH 5.8) at 7:00 am and 6:00 pm each day.

**Table 2:** Composition and nutrient analysis of the experimental diet (as‐fed basis)

Ingredients	Percentage (%)	Nutrient analysis	
Corn	65.00	DE (MJ kg^−1^)	13.35
Soybean meal	22.00	CP (%)	16.80
Wheat bran	9.25	NDF (%)	11.88
Soybean oil	0.70	ADF (%)	4.12
Lys	0.18		
Thr	0.01		
CaHPO_3_	0.69		
Rock powder	0.87		
Salt	0.30		
1% Premix^a^	1.00		

ADF, acid detergent fiber; CP, crude protein; DE, digestible energy; NDF, neutral detergent fiber

a Premix provided these amounts of vitamins and minerals per kilogram on an as‐fed basis: vitamin A, 10,800 IU; vitamin D3, 4,000 IU; vitamin E, 40 IU; vitamin K3, 4 mg; vitamin B1, 6 mg; vitamin B2, 12 mg; vitamin B6, 6 mg; vitamin B12, 0.05 mg; biotin, 0.2 mg; folic acid, 2 mg; niacin, 50 mg; D‐calcium pantothenate, 25 mg; Fe, 100 mg as ferrous sulfate; Cu, 150 mg as copper sulfate; Mn, 40 mg as manganese oxide; Zn, 100 mg as zinc oxide; I, 0.5 mg as potassium iodide; and Se, 0.3 mg as sodium selenite.

### Sample collection

At day 28, all pigs were euthanized, and the samples were collected. The colonic digesta was collected in a sterile tube and immediately stored at −20°C for the analysis of gut microbial composition and microbial metabolites. Furthermore, a small section of the proximal colon tissue was clipped and washed with phosphate buffer solution (PBS, pH 7.0), and the epithelial mucosa was then collected using a glass microslide, immediately frozen in liquid nitrogen and used to determine the expression of inflammatory cytokine‐related genes.

### Analysis of colonic microbial metabolites

0.4 g of colonic digesta was weighted into a 2 ml sterile centrifuge tube, and was added 1.5 ml of double distilled water for analysing SCFAs. The mixture was vortexed and centrifuged at 13 400 *g* for 10 min. Then, 1 ml supernatant was transferred into a new 2 ml centrifuge tube with adding 200 μl of 25% (w/v) metaphosphoric acid. After overnight at −20°C, the supernatant was centrifuged at 13 400 *g* for 10 min, then, filtered with a 0.22 μm filter for measurement. The Agilent 7890B Gas Chromatograph was used to determine the concentration of SCFAs according to the method by Wang and colleagues (2011).

Using high‐performance liquid chromatography (HPLC), the biogenic amines were measured by referring the method of Yang and colleagues (2014). 0.6 g of colonic digesta was weighted into a 2 ml centrifuge tube with adding to 1.5 ml trichloroacetic acid solution to precipitate the proteins and peptides. After extraction by n‐hexane, samples were derived using dansyl chloride. Gradient elution of two solvents was used as follows: solvent A consisted of HPLC grade water and solvent B was acetonitrile and the flow rate was 1.0 ml min^−1^. The wavelength of ultraviolet detector was 254 nm, and the column temperature was 30°C.

### DNA extraction and Illumina MiSeq sequencing

Microbial DNA extraction was carried out from the colonic digesta using TIANamp Stool DNA Kit (Tiangen, Beijing, China) according to the manufacturer's instructions. DNA concentrations were measured by a Nano‐Drop 1000 spectrophotometer (Thermo Scientific Inc, Wilmington, DE, USA). The V3‐V4 region of the bacterial 16S rRNA gene was amplified by PCR using primers 314F (5′‐CCTACGGGNGGCWGCAG‐3′) and 805R (5′‐GACTACHVGGGTATCTAATCC‐3′) (Guo *et al*., 2017). The PCR conditions and reaction mixtures were completed according to methods previously described (Cheng *et al*., 2017). The amplified production was sequenced on an Illumina MiSeq platform according to the standard protocols (Annoroad Gene Technology Co., Ltd., Beijing, China).

### Bioinformatics analysis

The raw sequences were processed using the MOTHUR software package (version 1.32.0) (Schloss *et al*., 2009). Gaps in each sequence were discarded in all samples to decrease noise with the screening, filtering, and pre‐clustering processes. Operational taxonomic units (OTUs) were clustered using the mean neighbour algorithm with a cut‐off of 97% similarity. Typical sequences from each OTU were taxonomically sorted with a confidence level of 90%, using the Ribosomal Database Project classifier (Cole *et al*., 2009). Spurious OTUs were removed and the remaining OTUs were used for estimation of species diversity.

The assessment of bacterial diversity included rarefaction analysis, a coverage estimator, an abundance‐based coverage estimator, a Chao richness estimator, and the Shannon and Simpson diversity index (Schloss *et al*., 2009). Principal coordinates analysis (PCA) from the MOTHUR output was performed using the unweighted distance method (Schloss *et al*., 2009). Significant and unique OTUs in each group were screened using a linear discriminant algorithm (LDA) effect size (LDA score > 2) (Segata *et al*., 2011).

### RNA extraction and real‐time PCR for genes related inflammatory cytokines

The RNA of colonic epithelial mucosa was extracted using RNApure Total RNA kit (Aidlab, Beijing, China) according to the manufacturer's instructions. The concentration of RNA was measured using a Nano‐Drop spectrophotometer (ThermoFisher Scientific, Wilmington, USA). The absorption ratios (OD260/OD280 nm) of samples were between 1.8 and 2.0, demonstrating the high purity of the RNA. The RNA concentration of all the samples was adjusted to one microgram per microlitre based on optical density. 1 μg RNA was reverse‐transcribed using a PrimeScript™ RT reagent Kit with gDNA Eraser (Takara Bio, Otsu, Japan) according to the manufacturer's instructions. Real‐Time quantitative PCR for the relevant genes was performed using correspondent complementary DNA and primers. The primers for genes related inflammation are listed in Table S2. Amplification was performed by the ABI 7300 Real‐Time qPCR system (Applied Biosystems, Foster, CA, USA) with fluorescence detection of SYBR green dye, the reaction conditions were previously described by Yu and colleagues (2017). The relative expression of genes was calculated using the $2^{-\Delta \Delta C_{t}}$ method.

### Statistical analysis

All data were analysed in SPSS 20.0 (SPSS Inc., Chicago, IL, USA) and graph generated using Graphpad Prism (La Jolla, CA, USA). Microbial data were analysed using Mann–Whitney *U*‐*test*. Genes related data were analysed using Student's *t*‐test. Data are showed mean ± SEM or medians, as indicated. Significant differences were set at *P *≤* *0.05, and tendency was declared with 0.05 < *P *<* *0.10.

## Conflict of interest

The authors declare that they have no conflict of interest.

## Supporting information


**Fig. S1.** The rarefaction curves in control group and propionate group.
**Table S1.** Top 30 predominant OTUs.
**Table S2.** The primers sequence.
