# Supplementary material for: Caecal infusion of the short‐chain fatty acid propionate affects the microbiota and expression of inflammatory cytokines in the colon in a fistula pig model
Source: Microb Biotechnol. 2018 Jun 1;11(5):859–68. doi: 10.1111/1751-7915.13282 (PMC6116746; doi:10.1111/1751-7915.13282)
Supplement: Supplementary file 1 — Fig. S1. The rarefaction curves in control group and propionate group. Table S1. Top 30 predominant OTUs. Table S2. The primers sequence. [file MBT2-11-859-s001.docx]

**Figure S1** The rarefaction curves in control group and propionate group


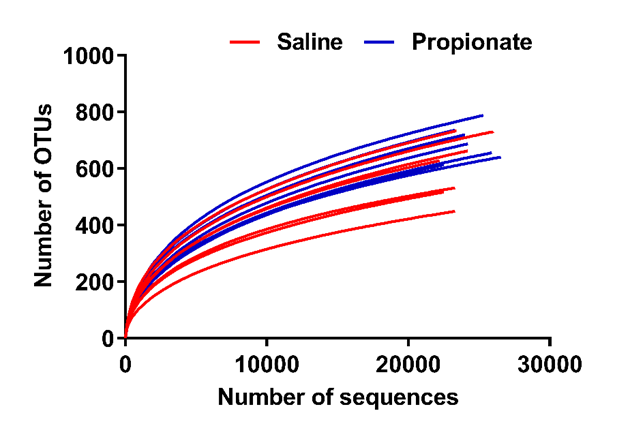


**Table S1** Top 30 predominant OTUs

| OTU ID | Saline | Propionate | *P*-value | Annotation^1^ |
| --- | --- | --- | --- | --- |
| 349024  807795  588197  581474  463794  780650  712677  553352  354905  752354  568118  4416659  345899  332718  584001  577228  29495  570507  1107027  620319  316587  560535  524884  367213  318764  370287  254011  1110312  315189  324283  354971  589114  509149  333178  347529 | 17.33±3.67  2.41±0.87  6.15±1.10  7.56±1.50  10.01±1.82  2.73±1.02  1.79±0.61  3.47±0.77  3.33±0.61  1.70±0.66  0.49±0.26  1.40±0.23  0.98±0.71  0.53±0.07  1.05±0.26  0.65±0.39  0.60±0.27  1.13±0.29  1.64±0.29  0.47±0.10  0.63±0.10  1.63±0.35  0.66±0.20  1.89±0.49  1.44±0.38  0.86±0.33  0.34±0.11  0.61±0.14  0.52±0.13  0.10±0.06  0.91±0.23  0.57±0.22  0.61±0.16  0.58±0.09  0.80±0.18 | 20.28±4.55  10.83±6.34  5.31±0.84  4.57±1.79  4.52±1.08  3.91±1.25  2.39±0.66  2.14±0.86  2.11±0.85  1.82±0.47  1.47±0.28  1.33±0.21  1.17±0.30  1.03±0.50  0.99±0.28  0.99±0.29  0.92±0.43  0.86±0.19  0.77±0.18  0.76±0.21  0.65±0.12  0.63±0.09  0.62±0.28  0.60±0.17  0.60±0.17  0.59±0.21  0.59±0.26  0.58±0.26  0.51±0.16  0.50±0.22  0.49±0.20  0.34±0.14  0.31±0.14  0.27±0.07  0.21±0.04 | 0.805  0.710  0.805  0.259  0.026  0.383  0.620  0.259  0.209  0.535  0.038  1.000  0.209  0.710  0.902  0.383  0.902  0.710  0.026  0.620  0.902  0.053  0.535  0.073  0.073  0.902  1.000  0.456  1.000  0.165  0.259  0.456  0.165  0.026  0.007 | g_ Streptococcus  g_ Lactobacillus  g_ Lactobacillus  g_ Lactobacillus  g_ Lactobacillus  f_ Clostridiaceae  g_ SMB53  g_ Lactobacillus  g_ Lactobacillus  g_ Coprococcus  s_ Prevotella _ copri  g_Lactobacillus  s_ Prevotella _ copri  g_ Lactobacillus  g_ Lactobacillus  f_ S24-7  f_ Ruminococcaceae  g_ Blautia  g_ Lactobacillus  o_ Clostridiales  g_ Streptococcus  f_ Ruminococcaceae  s_ biforme  f_ Ruminococcaceae  g_ Lactobacillus  s_ Prausnitzii  g_ Lactobacillus  o_ Clostridiales  g_ Lactobacillus  g_ Prevotella  g_ Lactobacillus  g_ Lactobacillus  g_ Lactobacillus  g_ Lactobacillus  g_ Turicibacter |

^1^s = species; g = genus; f = family; o = order.

**Table S2** The primers sequence

| Genes | Forward primer (5’-3’) | Reverse primer (5’-3’) | Reference^1^ |
| --- | --- | --- | --- |
| NF-κB | AACCCCTTCCAAGTTCCCA | TCCCCGAGTTCCGATTCAC | (Zheng *et al*., 2012) |
| HDAC1 | GTTGGAAGGGCTGATGTG | TGCTCGCTGCTGGACTTA | (Liu *et al*., 2012) |
| CXCL1 | GCACGCTGTACCATCCACTG | CATACAACCCCCCCTACCCT | (Sargeant *et al*., 2010) |
| CXCL2 | TGCAGACCGTGCAAGGAATT | TGGCTATGACTTCCGTTTGGT | (Sargeant *et al*., 2010) |
| MCP-1 | TCACCAGCAGCAAGTGTCCT | ATGTGCCCAAGTCTCCGTTT | (Villodre Tudela *et al*., 2015) |
| IL-2 | TCTTGTGTTGCATTGCACTAA | TCAGAGTTTTTGCTTTGACCTAA | (Suradhat *et al*., 2003) |
| IL-6 | CCTCTCCGGACA AAACTGAA | TCTGCCAGTACCTCCTTGCT | (Feng *et al*., 2015) |
| IL-8 | TAGGACCAGAGCCAGGAAGA | AGCAGGAAAACTGCCAAGAA | (Feng *et al.*, 2015) |
| IL-1β | AGTGGAGAAGCCGATGAAGA | CATTGCACGTTTCAAGGATG | (Feng *et al*., 2015) |
| IL-18 | TATGCCTGATTCTGACTGTT | ATGAAGACTCAAACTGTATCT | (Pie *et al*., 2004) |
| IL-10 | CTGCCTCCCACTTTCTCTTG | TCAA AGGGGCTCCCTAGTTT | (Feng *et al*., 2015) |
| TNF-α | CCACGCTCTTCTGCCTACTGC | GCTGTCCCTCGGCTTTGAC | (Feng *et al*., 2015) |
| IFN-γ | TCCAGCGCAAAGCCATCAGTG | ATGCTCTCTGGCCTTGGAACATAGT | (Villodre Tudela *et al*., 2015) |
| β-actin | ATGCTTCTAGACGGACTGCG | GTTTCAGGAGGCTGGCATGA | (Lin *et al*., 2014) |

^1^Reference

Feng, Z.M., Li, T.J., Wu, C.L., Tao, L.H., Blachier, F., and Yin, Y.L. (2015). Monosodium L-glutamate and dietary fat exert opposite effects on the proximal and distal intestinal health in growing pigs. *Appl Physiol Nutr Me* **40:** 353-363.

Lin, M., Zhang, B., Yu, C., Li, J., Zhang, L., Sun, H., *et al*. (2014). L-Glutamate supplementation improves small intestinal architecture and enhances the expressions of jejunal mucosa amino acid receptors and transporters in weaning piglets. *PLoS One* **9:** e111950.

Liu, L., Liu, Y., Gao, F., Song, G., Wen, J., Guan, J., *et al*. (2012). Embryonic development and gene expression of porcine SCNT embryos treated with sodium butyrate. *J Exp Zool B Mol Dev Evol* **318:** 224-234.

Pie, S., Lalles, J.P., Blazy, F., Laffitte, J., Seve, B., and Oswald, I.P. (2004). Weaning is associated with an upregulation of expression of inflammatory cytokines in the intestine of piglets. *J Nutr* **134:** 641-647.

Sargeant, H.R., McDowall, K.J., Miller, H.M., and Shaw, M.A. (2010). Dietary zinc oxide affects the expression of genes associated with inflammation: Transcriptome analysis in piglets challenged with ETEC K88. *Vet Immunol Immunopathol* **137:** 120-129.

Suradhat, S., Thanawongnuwech, R., and Poovorawan, Y. (2003). Upregulation of IL-10 gene expression in porcine peripheral blood mononuclear cells by porcine reproductive and respiratory syndrome virus. *J Gen Virol* **84:** 453-459.

Villodre Tudela, C., Boudry, C., Stumpff, F., Aschenbach, J.R., Vahjen, W., Zentek, J., *et al*. (2015). Down-regulation of monocarboxylate transporter 1 (MCT1) gene expression in the colon of piglets is linked to bacterial protein fermentation and pro-inflammatory cytokine-mediated signalling. *Br J Nutr* **113:** 610-617.

Zheng, R., Yang, L., Zhou, X.L., Zhu, C., Shu, X.G., Wu, X., *et al*. (2012). Effect of soybean oligosaccharides on immunity and TLR2-NF-κB signal pathway response for weanling pigs. *Journal of Food Agriculture & Environment* **10:** 273-279.
